# Intra-articular injection of epigallocatechin (EGCG) crosslinks and alters biomechanical properties of articular cartilage, a study via nanoindentation

**DOI:** 10.1371/journal.pone.0276626

**Published:** 2022-10-25

**Authors:** Mary Pat Reiter, Shawn H. Ward, Barbara Perry, Adrian Mann, Joseph W. Freeman, Moti L. Tiku

**Affiliations:** 1 Department of Biomedical Engineering, Rutgers University, Piscataway, New Jersey, United States of America; 2 Department of Materials Science and Engineering, Rutgers University, Piscataway, New Jersey, United States of America; 3 Department of Orthopedic Surgery, Rutgers University Robert Wood Johnson Medical School, New Brunswick, New Jersey, United States of America; 4 Department of Medicine, Robert Wood Johnson Medical School, New Brunswick, New Jersey, United States of America; Drexel University, UNITED STATES

## Abstract

Osteoarthritis and rheumatoid arthritis are debilitating conditions, affecting millions of people. Both osteoarthritis and rheumatoid arthritis degrade the articular cartilage (AC) at the ends of long bones, resulting in weakened tissue prone to further damage. This degradation impairs the cartilage’s mechanical properties leading to areas of thinned cartilage and exposed bone which compromises the integrity of the joint. No preventative measures exist for joint destruction. Discovering a way to slow the degradation of AC or prevent it would slow the painful progression of the disease, allowing millions to live pain-free. Recently, that the articular injection of the polyphenol epigallocatechin-gallate (EGCG) slows AC damage in an arthritis rat model. It was suggested that EGCG crosslinks AC and makes it resistant to degradation. However, direct evidence that intraarticular injection of EGCG crosslinks cartilage collagen and changes its compressive properties are not known. The aim of this study was to investigate the effects of intraarticular injection of EGCG induced biomechanical properties of AC. We hypothesize that in vivo exposure EGCG will bind and crosslink to AC collagen and alter its biomechanical properties. We developed a technique of nano-indentation to investigate articular cartilage properties by measuring cartilage compressive properties and quantifying differences due to EGCG exposure. In this study, the rat knee joint was subjected to a series of intraarticular injections of EGCG and contralateral knee joint was injected with saline. After the injections animals were sacrificed, and the knees were removed and tested in an anatomically relevant model of nanoindentation. All mechanical data was normalized to the measurements in the contralateral knee to better compare data between the animals. The data demonstrated significant increases for reduced elastic modulus (57.5%), hardness (83.2%), and stiffness (17.6%) in cartilage treated with injections of EGCG normalized to those treated with just saline solution when compared to baseline subjects without injections, with a significance level of alpha = 0.05. This data provides evidence that EGCG treated cartilage yields a strengthened cartilage matrix as compared to AC from the saline injected knees. These findings are significant because the increase in cartilage biomechanics will translate into resistance to degradation in arthritis. Furthermore, the data suggest for the first time that it is possible to strengthen the articular cartilage by intraarticular injections of polyphenols. Although this data is preliminary, it suggests that clinical applications of EGCG treated cartilage could yield strengthened tissue with the potential to resist or compensate for matrix degradation caused by arthritis.

## I. Introduction

The most common forms of arthritic disorders are osteoarthritis (OA) and rheumatoid arthritis a degenerative and inflammatory arthritis, respectively. In OA, the disease pathology initiates in the articular cartilage component of the joint [1]. Because of the unique structure of the articular cartilage matrix, particularly its collagen component when destroyed cannot be rebuilt while loss of proteoglycan component is replaced [2–4]. OA is one of the leading causes of pain, loss of function, and disability in adults in the US alone, this affects more than 30 million people [5, 6]. Rheumatoid Arthritis (RA) like OA, contributes to a burden of disability. To date, no pharmacological intervention offers protection or treatment from the destruction of articular cartilage.

Articular cartilage is a dense connective tissue composed of predominantly type II collagen crosslinked fibers and within it are embedded proteoglycans, which provide compressive properties, and the chondrocytes. On the other hand, crosslinked collagen provides tensile properties of articular cartilage. Cartilage is an avascular tissue, so chondrocytes obtain their nutrients through diffusion. Articular cartilage serves several functions. It provides a smooth surface for the movement of articulating bones and helps in shock absorption and tensile strength for joint load and mobility between neighboring bones. Human AC has an elastic compressive modulus ranging from 240–1000 kPa [7–10] and a tensile modulus of 5–25 MPa [11–13]. Its composition differs with age, site in the joint, and depth from the surface. Normal AC contains 60 to 70% collagen and 5 to 15% proteoglycans on a dry weight basis. It is divided into various zones, the superficial zone, intermediate, deep zone, and calcified zone [14].

When arthritic AC experiences reduced integrity and mechanical properties that can lead to further damage [15] resulting in bone damage and increases the risk of further joint injury [16–18]. As AC has limited repair characteristics, surgical intervention such as matrix-induced autologous chondrocyte implantation (MACI), chondrocyte seeded scaffolds, and hyaluronic-acid based scaffolds have been used to treat significant degradation [19]. These surgical treatments, however, are imperfect, carry risks, and do not help in AC repair. Consequently, researchers have sought less invasive options and preventative measures.

In vitro assessing mechanical function of articular cartilage provides a readout of crosslinking and compressive properties of the matrix tissue. One such test for assessing AC condition is nanoindentation. Indentation is a mechanical test during which a sharp tip is pushed into a surface to produce deformation while recording the displacement of the tip into the surface and the force exerted on the tip at given time intervals [20]. These values are plotted to make load-displacement curves which are then used to calculate hardness, reduced elastic modulus, and stiffness based on semi-empirical curve fitting and elastic contact theory [21]. The viscoelasticity of cartilage requires a more specialized method of indentation. Previous indentation studies of AC have removed it from the bone, opted for micro-indentation, tested the cartilage cross-section, or used large animal samples [13, 20]. Each of these would contribute to significant errors. Furthermore, cartilage’s complex structure could also mean these methods will fail to give an accurate measurement of the overall properties due to the unnatural test setting. Thus, it is crucial to have a reliable and sensitive method of testing mechanical functions that would reflect in-vivo conditions of articular cartilage. Therefore, in this study, we developed a mounting method for intact articular cartilage attached to subchondral bone at the end of the joint.

In arthritis, there is enhanced production and activation of aggrecan-degrading enzyme disintegrin, metalloproteinase with thrombospondin motif (ADAMTSs) and matrix metalloproteinases (MMPs) resulting in degradation of aggrecan and type II collagen of articular cartilage, respectively [22]. Arthritis degrades AC, impairs mechanical properties, and prevents the tissue’s ability to absorb shock, lubricate movement, and protect the underlying bone. Based on this premise inhibitors to ADAMTSs and MMPs were thought to have a promise of reducing cartilage or joint degradation. However, to date, the promise has not come to clinical fruition [23]. Taking clues from antiquity practice of leather tanning, a process of the conversion of skin/hide (type I collagen) matrix into leather through the crosslinking of plant polyphenols (tannin) with Type I collagen matrix, the role of crosslinking articular cartilage with polyphenols was investigated by our group [24].

In our study in-vitro articular cartilage tissue samples treated with various polyphenols were shown to crosslink cartilage making it resistant to collagenase treatment. In addition, for example, EGCG treatment showed a maximum increase in the thermal stability of cartilage by 12°C [24]. Enhancement of thermal stability indicates binding of polyphenols with collagen in the cartilage matrix, as well as collagen cross-linking. As type I and type II collagen are structurally similar we can infer that the mechanism of crosslinking by polyphenols in type II collagen crosslinking is similar to type I collagen. Prophylactic and therapeutic intra-articular injections of polyphenols such as EGCG in rat inflammation model of arthritis study also showed significant prevention of articular cartilage damage in treated versus in the controls [24]. However, in the study, it was not shown if an intra-articular injection of polyphenols changes the biomechanical properties of articular cartilage. Thus, further investigations are warranted to move this area of scientific study to clinical fruition. With that goal, in the present studies, we set to investigate changes in the biomechanical properties of polyphenol injected joint cartilage. We propose EGCG treatment strengthens the articular cartilage by crosslinking and making it resistant to degradation and withstand mechanical forces in the joint. An evaluation by the method of nanoindentation measuring the hardness, reduced elastic modulus and stiffness of EGCG intraarticular compared to the control injected joint AC will provide direct proof of the principle that crosslinking AC is a mechanistically and scientifically sound option. Increased mechanical properties will potentially decrease the damage caused by the pathways of cartilage and joint destruction.

## II. Materials and methods

### II.I Materials

EGCG (-)-Epigallocatechin-3-gallate (≥95%) was purchased from Sigma-Aldrich (St. Louis, MO, USA). Phosphate-buffered saline (PBS) was made at Rutgers University. For nanoindentation, a Triboindenter 950 with a Ti-0053 Diamond, Berkovich, 100nm fluid cell probe was kindly provided by Rutgers University, Material Science and Engineering, both manufactured by Bruker (Billerica, MA, USA). Clear super glue gel was purchased from Gorilla Glue (Sharonville, OH, USA). Quick-setting steel-reinforced epoxy was obtained from J-B Weld (Atlanta, GA, USA). Polystyrene tissue culture 15 ml tubes with screw caps were obtained from Corning (Corning, NY, USA). Surgical steel earring posts 6mm and steel thumbtacks were obtained from Darice (Strongsville, OH, USA) and a local home improvement store (Highland Park, NJ, USA), respectively. Plain microscope slides and #10 and #11 stainless steel scalpel blades were obtained from Fisher Scientific (Pittsburgh, PA, USA). #12 stainless steel scalpel blades were purchased from Surgical Design (Armonk, NY, USA). Microdissection scissors manufactured by Fine Science Tools were used (Foster City, CA, USA). A precision pin hand drill was purchased from Autotoolhome (Shenzhen, China). A high-speed rotary tool 300 series with new 1.5” fiberglass reinforced rotary cut-off wheels 456 series manufactured by Dremel were used for sample preparation (Mt. Prospect, IL, USA).

### II.II Ethics statement

All animal experiments were carried out in compliance with Rutgers University Institutional Animal Care and Use of Committee (IACUC). The protocols were approved by the Rutgers University IACUC, PROTO999900329 and PROTO999900328.

### II.III Injection treatment

#### EGCG treatment for histological study

One rat underwent no injection. Two rats underwent EGCG injection and control saline as per the protocol. The harvested joints were formalin fixed. Treatment samples were processed at Bolder BioPATH. Preserved and decalcified (5% formic acid) knee joints were cut in half in the frontal plane, processed through graded alcohols and a clearing agent, infiltrated, and embedded in paraffin, sectioned, and stained with toluidine blue by Bolder BioPATH, Inc. associated personnel (HistoTox Labs, Inc.). Tissues were examined microscopically by a board-certified veterinary pathologist (Dr. Alison Bendele) and observations were entered into a computer-assisted data retrieval system. Wherever possible, histologic changes were described according to their distribution, severity, and morphologic character. Description and classification of lesions commonly seen in laboratory animals were by accordance with Jubb et al., 1993 [25].

#### EGCG treatment for nanoindentation study

The injection protocol for these studies is based on our previous study investigating the effects of EGCG on inflammation in rat model [24]. Six female Lewis rats were used for this study each weighing approximately 350 g, the groups are described in Table 1. Three of the rats underwent a 25μL injection of 1.2% wt/v EGCG in saline in their right knee and a 25μL injection of saline in their left knee. The injections were performed daily for five days on the lateral side of the knees. The specifics of this protocol were created to prevent clearance of the EGCG from the joint cavity and is based on our previous in vivo study with polyphenol injections [24]. Three other rats underwent no injections and were used as a baseline control to normalize values. Knees were harvested post-mortem, severed at the midpoint of the femur and the midpoint of the fibula and tibia. Specimens were kept frozen at -20°C until testing preparation.

**Table 1:** Treatment groups of nanoindentation study.

	Left limb treatment	Right limb treatment
**Experimental**	25μL saline	1.2% wt/v EGCG in 25μL saline
**Control**	No injection	No injection

### II.IV Sample preparation and mount prototype

Two nights prior to planned indentation, a sample contained in a test tube would be moved to 4-degrees Celsius for a slow thaw. The day before planned indentation, the lower leg of the sample was carefully separated from the femur with care to not damage the AC. The fibula was removed. The tibias were cut to fit upright within the test tube using a Dremel and a Dremel 456 1.5” reinforced rotary cut-off wheel. The marrow was removed by rinsing with PBS using a syringe until clear. For smaller samples or cavities, a hand-drill was used to enlarge the space. After allowing the cavities to dry, a thumbtack for large cavities or an earring back for narrow cavities was inserted with super glue into the bone cavity and allowed to dry for 10 minutes. During this time, the samples were suspended inverted in PBS to maintain hydration.

**Fig 1:**
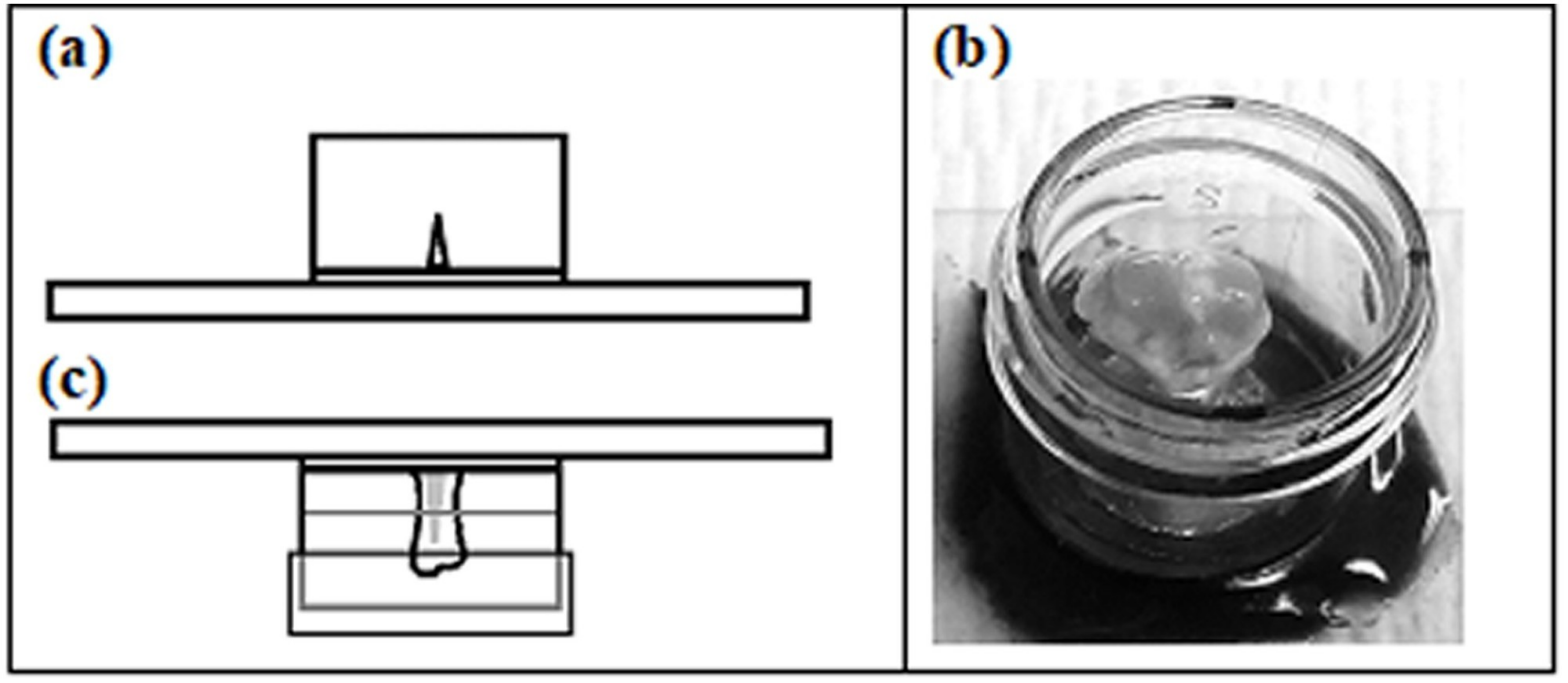
**(A-C).** Mounted tibial cartilage: (A) test-tube and earring back or thumbtack on microscope slide, (B) photograph of mounted tibia, (C) PBS emersion and mount inversion for storage.

To create the mount, approximately 2 cm of the top of a 15ml polystyrene test tube was sawed off. This was adhered to a glass microscope slide with quick-setting two-part epoxy and let set for 20 minutes as shown in Fig 1A. The thumbtack or earring back on which the sample was glued was then adhered to the microscope slide inside the test tube using JB-Kwik and allowed to set as shown in Fig 1B. After curing, the tubes were rinsed with PBS to remove any residue while avoiding the cartilage. They were then filled halfway with PBS, had their lids placed, inverted to ensure emersion of the cartilage in the fluid, and placed into a 4-degree Celsius refrigerator as shown in Fig 1C. Indentation was performed the following day.

### II.V Nanoindentation

**Fig 2:**
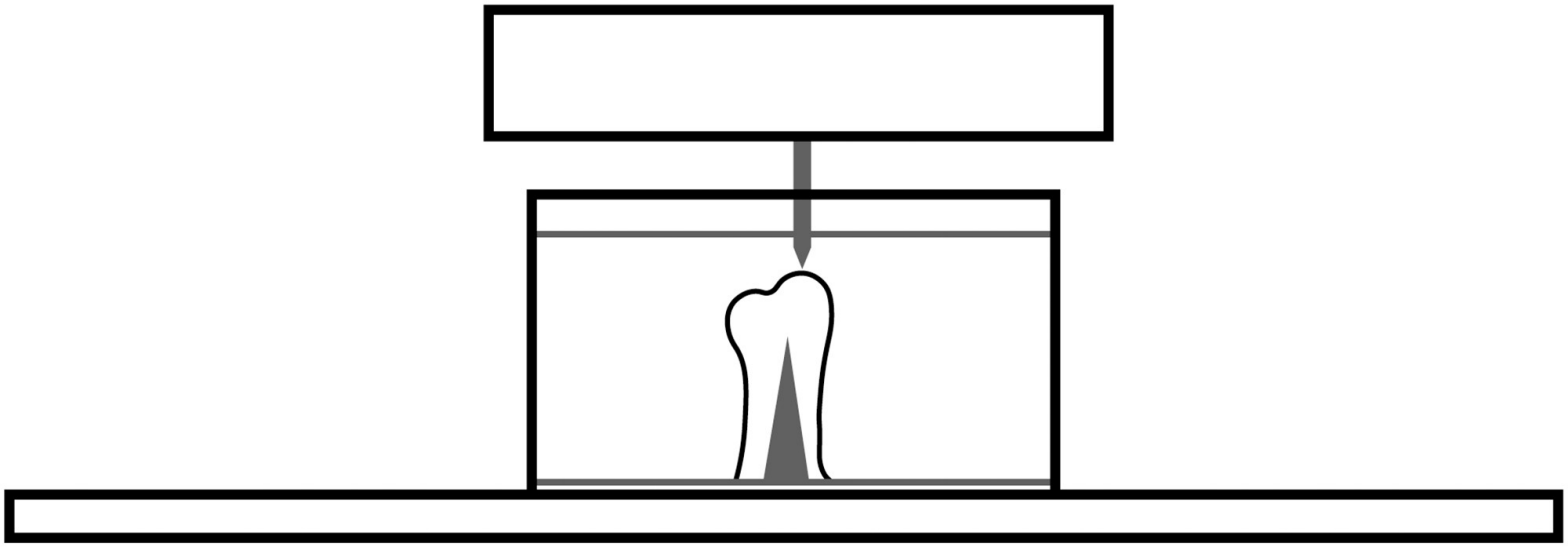
The diagram shows the location of the transducer and tip with respect to the mount’s tube lip, the sample, and the PBS level during sample evaluation.

Samples were flipped right-side up so that the microscope slide lay flat on the surface of the indenter stage. The remaining PBS was removed, and the sample temporarily dried during calibration. Calibration and testing were performed at room temperature (20**°C). Samples reached thermal equilibrium with the ambient air temperature.** Advanced Z-Axis Calibration needed to be performed first with the tip in the air, then with the tip submerged in PBS. A smaller test tube apparatus without a sample was made to simplify the process of PBS calibration. Typical procedures for Advanced Z-Axis Calibration would work for air. After setting safety limits around the lip of the test tube to ensure the tip would not collide with the tube, the tip was positioned above and then lowered approximately 2-mm into the fluid. Normal calibrations as if in air were then performed, and the tip was removed from the fluid and the calibration test-tube was removed from the machine. The test-tube with the sample was filled with PBS and loaded into the machine as shown in Fig 2.

**Fig 3:**
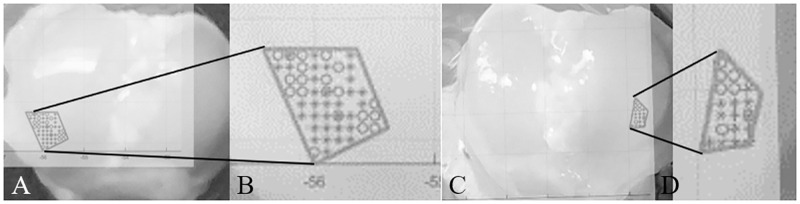
**A-D.** Photographs of the exposed tibia are shown overlaid with MATLAB graphs of X-Y stage coordinates indicating the locations of the indentations for Subject A’s (A) left knee joint, (B) left knee joint enlarged, (C) right knee joint, and (D) right knee joint enlarged.

**Fig 4:**
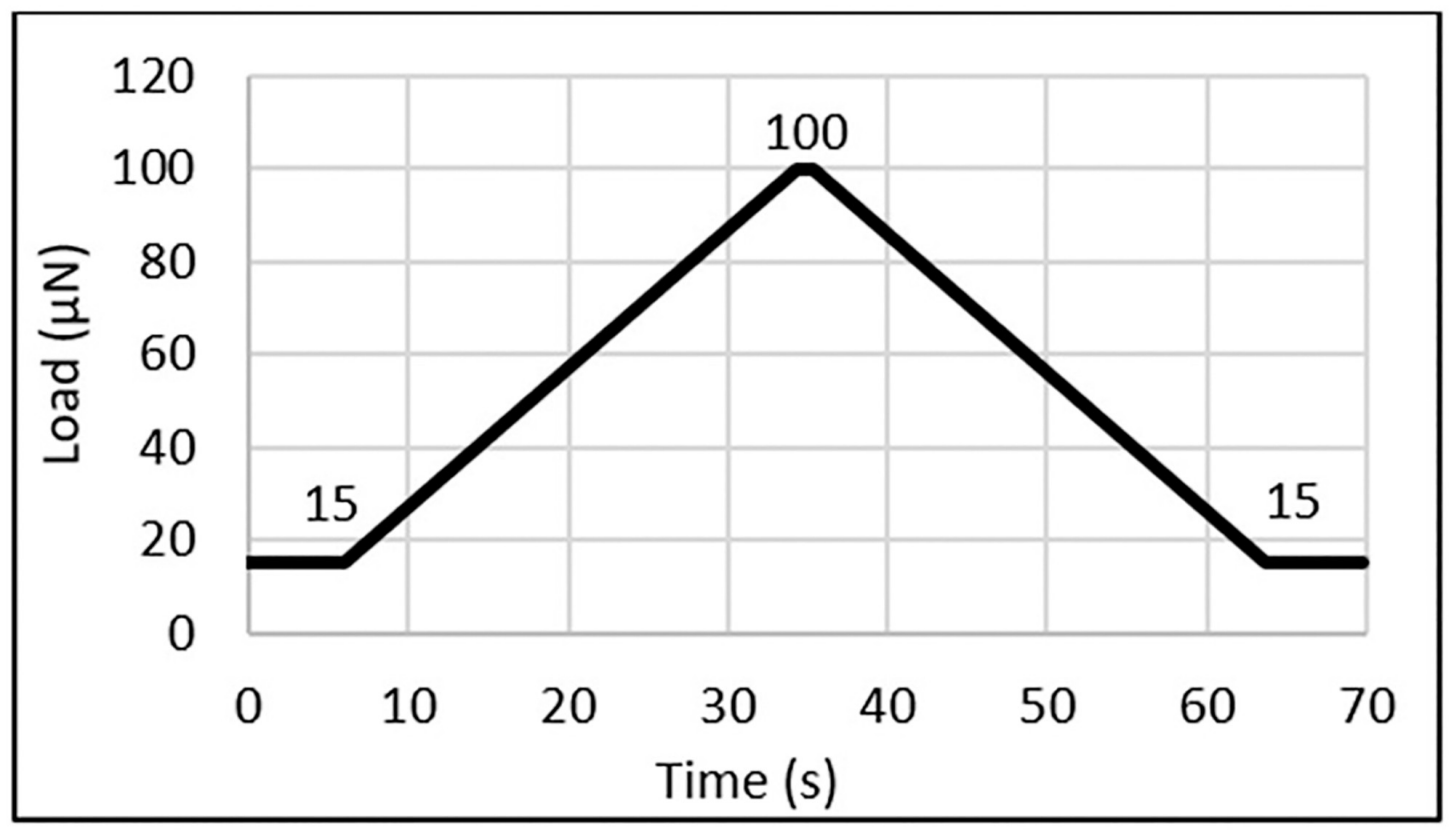
The plot shows the nanoindentation load function used for testing the samples where the y-axis is the load, and the x-axis is elapsed time during the test.

After determining indentation locations, indentation began. A variety of lattice separations were attempted with the most success being a grid with 200-micron separation. Specific regions of the samples were tested on each knee to ensure uniformity. 24–48 load-controlled indents were performed on the lateral most side of the lateral condyle. This ensured that differences across the full condyle were excluded for this study. The comparison was done by overlaying the MATLAB (MathWorks) plots with landmarks created on the test tube lips. Indentations were separated into two categories: failed and successful. Fig 3A–3D shows indentation locations for the left and right knee of Subject A with close-ups, respectively, where circles indicate failure and plus-signs indicate success. Failed indentations were typically due to variation in the height of the plateau of the sample which resulted in significant drift errors. Calculated positions were then refined from the larger data set to match successful locations on the respective opposite knee. The load function used was load-controlled with a preload of 15 μN to ensure the tip is in contact with the surface, maximum load of 100μN, 10 seconds of loading, 1 second hold at the maximum load, and 10 seconds of unloading as shown in Fig 4.

### II.VI Property calculations

Elastic Modulus (E_r_), Hardness (H), and Stiffness (S) for each indentation were calculated from the TriboIndenter software using Oliver-Pharr indentation analysis as shown in Eqs 1–3, respectively [26]. Though the Hertz Model is often used for calculating the mechanical properties of viscoelastic materials, the unusual loading curve prohibited accurate fitting. The Oliver-Pharr method calculates the properties based on the unloading curve which was more typical. The probe used had a radius of 100 nm which is sufficiently small to suggest that there would be minimal differences between values calculated using the Oliver-Pharr method and the Hertz Model [27, 28].

$$E_{r}=\frac{\sqrt{\pi }S}{2\beta \;\sqrt{A_{p}(h_{c})}}$$ (1)

$$H=\frac{P_{max}}{A_{r}}$$ (2)

$$S=2E_{r}\sqrt{\frac{P_{max}}{H\pi }}$$ (3)

where β is a constant for the material, A_p_(h_c_) is the projected contact area at contact depth h_c_, P_max_ is the peak applied load, and A_r_ is the residual indentation area.

Given the time-dependent mechanical properties of cartilage, the measured E_r_, H, and S are not entirely accurate quantitative values as the analysis assumes quasi-static conditions. However, they provide good relative values of the properties for the control, treated, and untreated cartilage.

### II.VII Statistical analysis

Raw values for each mechanical property outside two standard deviations of each mean were considered outliers and removed from calculations. An indentation was considered an outlier if any of the three properties were outside of the two standard deviations from the mean. The data was assessed in two ways.

The first method compares the results from all the specimens that received each treatment by combining it into four groups: Saline left limb, EGCG-treated right limb, Control left limb, and Control right limb (n = 3 for each). Recall that there were two groups: Group A was the specimens with a left-limb saline injection and a right-limb EGCG injection while Group B was the specimens that received no injections in either of their limbs. A one-way ANOVA with post-hoc Tukey test was run on these four groups with a significance value of 0.05.

The second method normalizes the results for each specimen and compares the ratio of change in each mechanical property for each specimen (n = 3 for each treatment, n = 6 for the entire study). The average of the right limb of each specimen was divided by the average of the left limb. A one-way ANOVA with post-hoc Tukey test was run on these two groups with a significance level of 0.05. By using normalized values, individual variation in specimen health or data collection has less likelihood of affecting the overall trend of the results. However, evaluating the trends for all of a certain subject group can provide information that may otherwise be lost.

Standard deviations are reported for the raw averages but are omitted from the normalized ratios given the effect that the individual standard deviations would contribute.

## III. Results

### Histomorphometry

**Fig 5:**
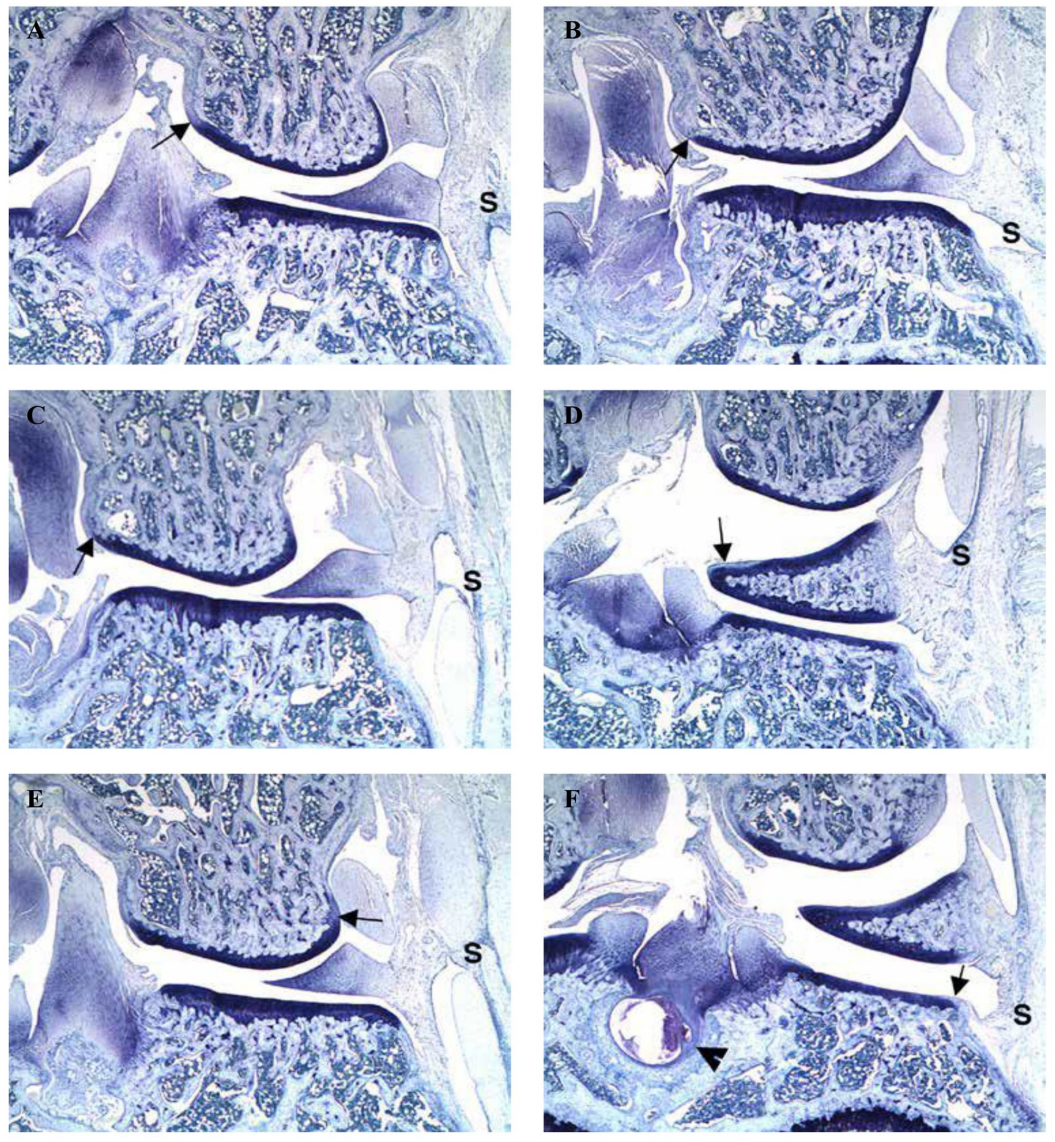
Histological images of cartilage at 25X. Figs A, C and E are right knee joints. Figs B, D, and F are the left knee joints. Figs A & B are from naïve rat. Figs C & E are from EGCG injected right knee joints and Figs D & F are from control saline-injected left knee joints. Knee shows very minimal multifocal synovial inflammation (S) with very minimal multifocal proteoglycan loss (arrow) of the marginal zone of the femur and tibia, as well as a bone cyst at the cruciate insertion (arrowhead).

In summary, all animals had very minimal inflammation of synovium, Table 2. Very minimal proteoglycan loss was seen in the marginal zone of the femur in all animals except MT-483, in the marginal zone of the tibia in animal MT-635, and in the lateral meniscus in animals MT-375 and MT-483. One animal (MT-635) had a bone cyst at the cruciate insertion. Collectively, there are no differences in histological observation between normal joints, EGCG, or saline injected joint samples, which suggests EGCG injection does not induce joint inflammation or articular changes indicating the non-toxic nature of this polyphenol (Fig 5).

**Table 2:** The qualitative results of the histological evaluations on individual animals. MT- 276 (right knee joint) and MT-291 (left knee joint) are from a single naïve rat. MT-375 and MT-516 are right knee joints injected with EGCG, and MT-483 and MT-635 are control saline-injected left knee joints from two rats.

Sample Name	Description
MT-276	Very minimal, multifocal inflammation of the synovium and very minimal, multifocal proteoglycan loss in the marginal zone of the femur.
MT-291	Very minimal, multifocal inflammation of the synovium and very minimal, multifocal proteoglycan loss in the marginal zone of the femur.
MT-375	Very minimal, multifocal inflammation of the synovium and very minimal, multifocal proteoglycan loss in the marginal zone of the femur and lateral meniscus.
MT-483	Very minimal, multifocal inflammation of the synovium and very minimal, focal proteoglycan loss in the lateral meniscus.
MT-516	Very minimal, multifocal inflammation of the synovium and very minimal, multifocal proteoglycan loss in the marginal zone of the femur.
MT-635	Very minimal, multifocal inflammation of the synovium and very minimal, multifocal proteoglycan loss in the marginal zone of the femur and tibia along with a bone cyst at the cruciate insertion.

### Nanoindentation

**Fig 6:**
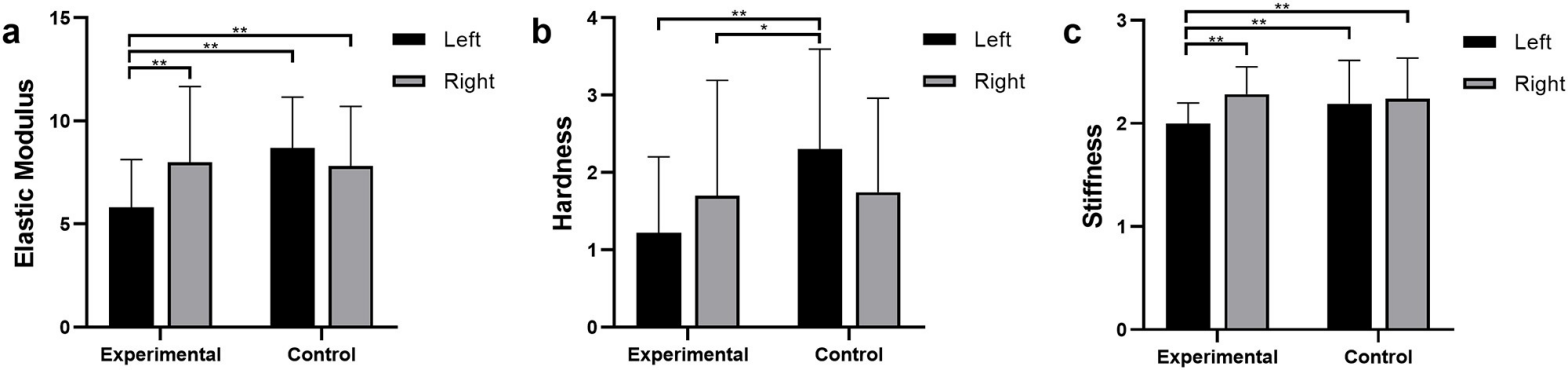
Bar charts indicating the raw averages and standard deviations for the four groups shown in Table 3. (*p < 0.05, ** p<0.01).

Calculations and comparisons were performed between the experimental and control groups for three mechanical properties: Reduced Elastic Modulus, Hardness, and Stiffness using Oliver-Pharr equations. The raw averages are shown in Table 3 and Fig 6. As described in statistical analysis, these values for combining all values from a specific treatment. The control group left and right limbs did not have statistically different values from each other, while the experimental group did for Elastic Modulus and Stiffness. There were statistical differences between the experimental left limb (saline injection) and the right and left limbs of the control groups for Elastic Modulus and Stiffness as seen in Fig 6.

**Table 3:** Results of nanoindentation as raw averages for the four groups of samples for three mechanical properties.

	Elastic modulus (MPa)	Hardness (MPa)	Stiffness (N/m)
**Experimental–Left, saline**	5.8 ± 2.3	1.2 ± 1.0	2.0 ± 0.2
**Experimental–Right, EGCG**	8.0 ± 3.7	1.7 ± 1.5	2.3 ± 0.3
**Control–Left, no injection**	8.7 ± 2.5	2.3 ± 1.3	2.2 ± 0.4
**Control–Right, no injection**	7.8 ± 2.9	1.7 ± 1.2	2.2 ± 0.4

**Fig 7:**
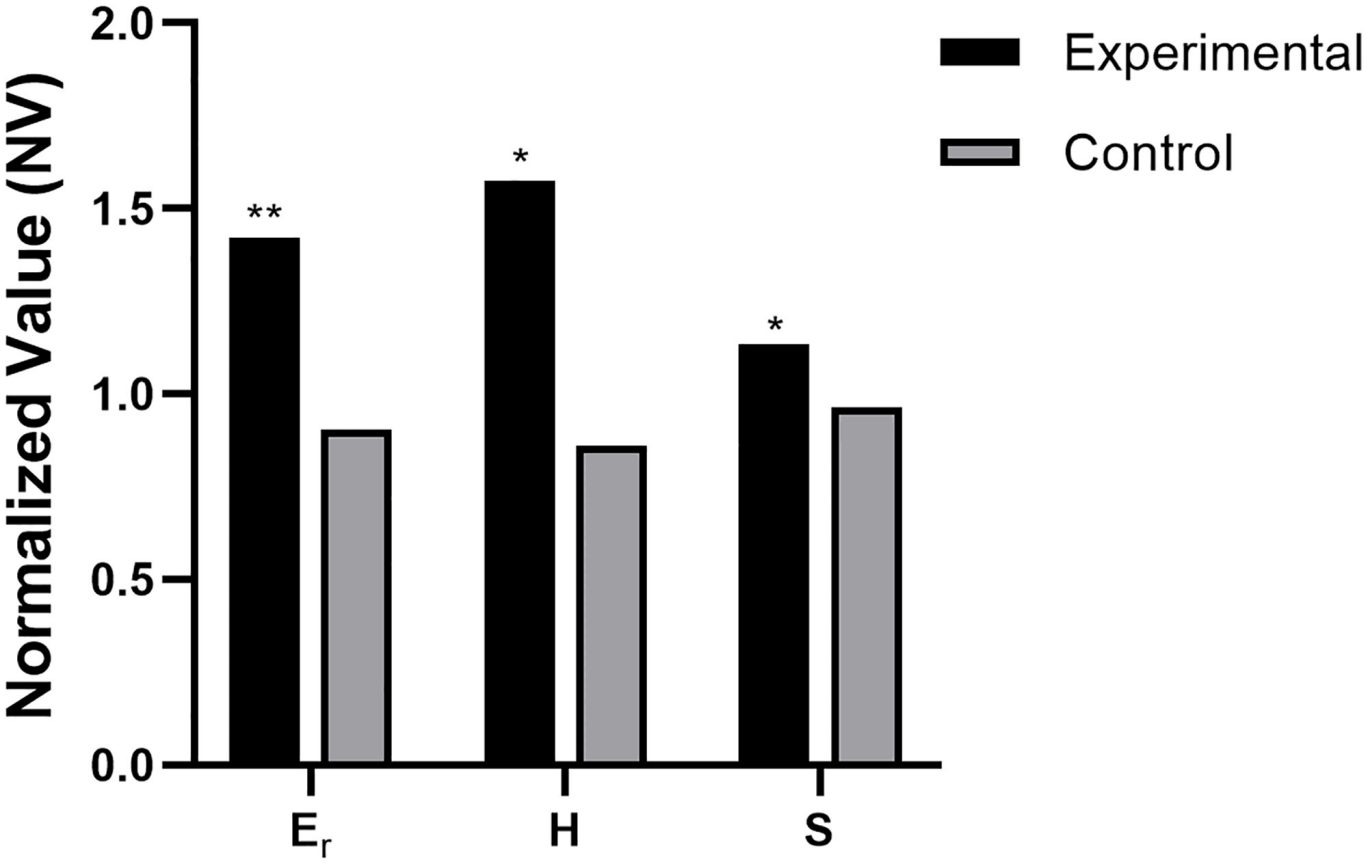
Normalized Value (NV) means (± standard deviation) for reduced elastic modulus, hardness, and stiffness comparing control specimen to treated specimen. Control NV is the mean of un-injected right knee joint divided by un-injected left knee joint. EGCG NV is the mean of EGCG-injected right knee joint divided by saline-injected left knee joint. NVs were calculated per each animal, then averaged for n = 3. (*p < 0.05, ** p<0.01).

For each specimen and each property, the normalized value (NV) was found by taking the average of the indentation values for the right knee divided by the average of the indentations for the left knee. This is not the average for all knees; this is the average of all indentations on one individual knee divided by the average of all of the indentations on the other individual knee. The normalized values are shown in Table 4. For all the mechanical properties, the NVs of the treated specimen were significantly greater than the NV of the control specimen as shown in Fig 7 based on a one-way ANOVA with post-hoc Tukey test.

**Table 4:** Results of the nanoindentation as normalized values for the two groups.

	Elastic modulus	Hardness	Stiffness
**Experimental (Right/Left)**	1.4	1.6	1.1
**Control (Right/Left)**	0.9	0.9	1.0
**% Increased**	57.5%	83.2%	17.6%
**P-value**	0.0039	0.0287	0.0324

**Fig 8:**
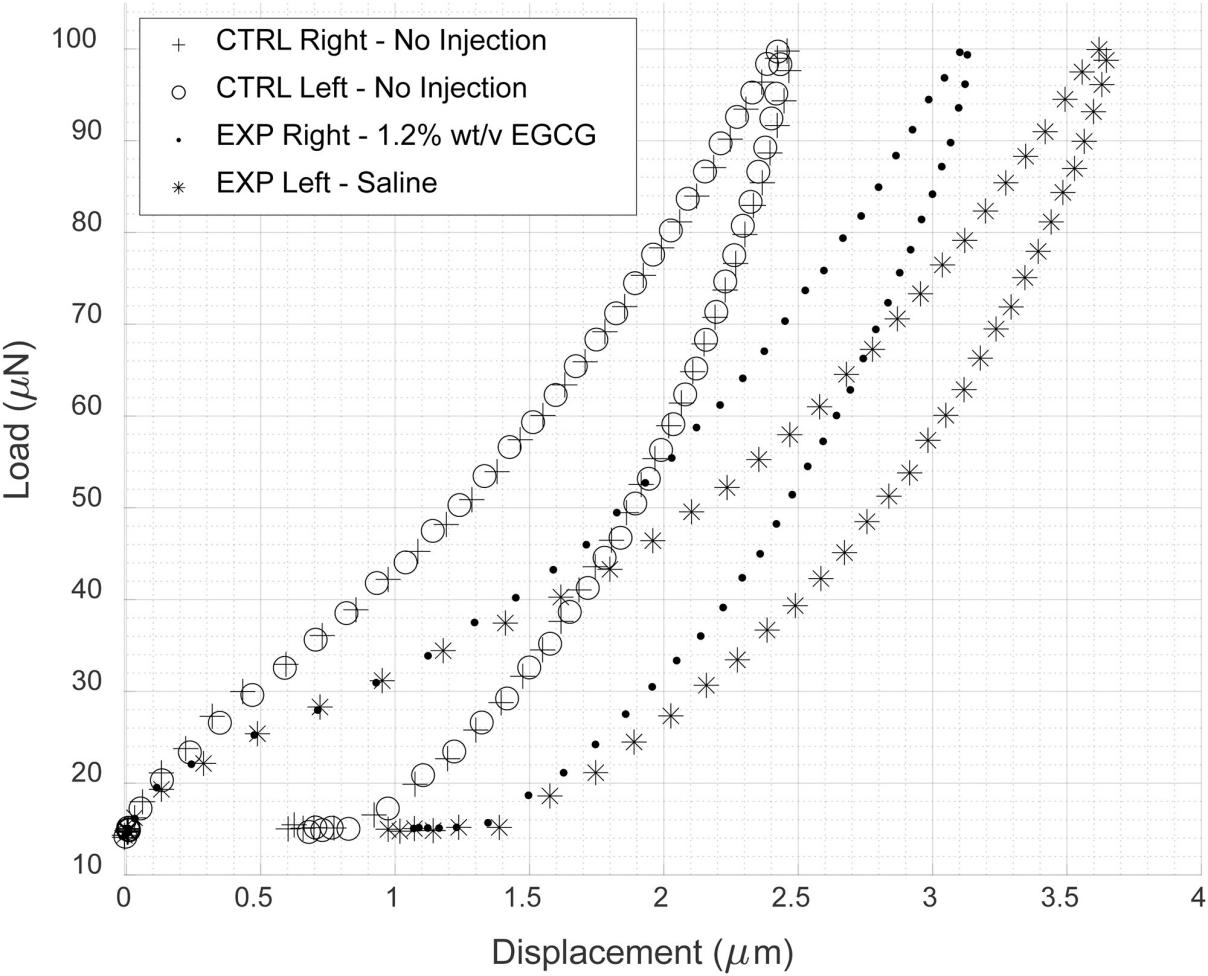
Load v displacement curves for four indentations. From a control animal shown is an un-injected right knee (+) and un-injected left knee (o). From an experimental animal shown is an EGCG-injected right knee (.) and saline-injected left knee (*).

The TriboIndenter software provides raw indentation information (time, load, displacement, x-y coordinates) in addition to calculated values of each mechanical property. The raw data is used by the software to produce Load vs Displacement curves from which the mechanical properties are calculated. A plot of four of these curves (right and left leg of a control sample and right and left leg of an experimental sample) is shown in Fig 8 in which every 60^th^ data point of the 4115 points of each curve was plotted. The unloading segment (lower portion) of each curve has drastically different slopes when comparing treated vs saline but relatively similar slopes when comparing no injections to no injections.

## IV. Discussion

There currently exists no pharmacological agent that can prevent or treat articular cartilage degradation. Persistence and progression of articular cartilage damage change mechanical forces within the joint leading to abnormal distribution of weight. These abnormalities trigger responses both in the articular cartilage and the bone (22). Since articular cartilage has limited regenerative capacity, cartilage degradation progression results in fissure, fragmentation and leads to total loss of articular cartilage [29].

The data presented in this manuscript demonstrates the ability of EGCG to alter cartilage in a way that will allow it to better withstand stress and loading through increases in modulus, stiffness, and hardness. These changes in the mechanical properties of EGCG injected joint cartilage indicate polyphenol mediated crosslinking of the collagen in cartilage, which can increase the longevity of the collagen in the matrix tissue [30]. This crosslinking makes the cartilage difficult to compress. However, arthritic joints are also subjected to a milieu of MMPs resulting in matrix degradation (17). In-vitro polyphenol treated articular cartilage has been shown to become resistant to collagenases, thus it is likely that polyphenol injected joint cartilage will have some resistance to enzymatic degradation (24). Polyphenol-mediated alteration of biomechanical properties and enhancement of enzymatic resistance together could provide a better way to resist cartilage degradation in arthritis. If arthritis has begun and the cartilage has been damaged, EGCG crosslinking may mechanically strengthen the tissue to help it resist mechanical stress and further degradation. EGCG in the synovium also will inhibit the inflammatory response, protecting the tissue from further degradation [31, 32].

These ideas are supported by our previous work which demonstrated a reduction in cartilage degradation in an in vivo arthritis model with in vivo injection of EGCG using histological imaging [24]. This would reduce the pain experienced by patients and delay the need for surgical intervention. The addition of EGCG may also serve as a preventative measure for high-risk individuals that still have normal cartilage. By mechanically strengthening the cartilage matrix tissue will increase its resistance to mechanical wear, potentially delaying the onset of diseases such as osteoarthritis. This form of treatment could significantly reduce the burden of disability caused by arthritis in the general population as well as the burden of long-term healthcare. These potential treatments would require exact dosing based on the amount of damage and the amount of crosslinking needed. Our previous work focused more on the effects of EGCG on inflammation and tissue degradation, while this study looks at the effect of EGCG on tissue mechanics.

Nanoindentation has been widely used to evaluate nano-to-micro level mechanical properties of samples ranging from materials for industrial applications to biological tissues. In biological tissues, specifically soft, viscoelastic materials, a primary benefit of nanoindentation is the minimally destructive nature of the test sample. Due to the complex nature of AC including the multiple layers and components, mechanical properties can vary based on the depth of testing. Maintaining a consistent method for testing across sample sets, as done here, is crucial in assessing cartilage health and creating a reproducible study tool for future investigations. This testing method, which includes the attachment to the subchondral bone, allows the tissue to be examined in a more natural setting. The entire knees are mounted in a saline solution and the cartilage is not removed from the bone, which limits any damage to the tissue that could alter the data.

It can be argued that an additional assessment of crosslinking aside from the evaluation of mechanical properties is necessary. In some studies tissue explants are subjected to thermal stability studies. Unfortunately, it is not possible to obtain a sufficient rat cartilage sample to perform thermal stability studies presently. Therefore, biomechanical physical properties are surrogate proof of additional crosslinking of cartilage by polyphenols.

In this study, mounting and testing methods for mechanically evaluating rat AC using nanoindentation was developed. Using this technique, the reduced elastic modulus, hardness, and stiffness of cartilage treated with EGCG, cartilage treated with saline, and cartilage left untreated were tested. Through nanoindentation, it was found that, when normalized, all three mechanical properties were increased in samples that were subjected to EGCG treatment compared to those that had not with a confidence interval of 95%, suggesting polyphenolic interaction with articular cartilage. Load vs displacement curves for the samples further show a difference in the samples; the EGCG-treated knees had a steeper unloading curve (which is related to contact stiffness) compared to their saline-treated counterpart; the left and right untreated knee indentations had identical unloading curves. This difference is likely due to EGCG initiated crosslinks. This observation from the raw data plots further supports the mechanical properties calculated in the results and indicates the robustness and sensitivity of this technique for future studies.

Articular cartilage is a porous matrix that facilitates the diffusion of small molecules such as EGCG. The injected polyphenol could traverse various layers of cartilage, where the hydroxyl functional groups of polyphenols (EGCG) can react with the side-chain functional groups of proline-rich collagens [33]. These interactions can strengthen the collagen and stabilize the cartilage matrix. Based on clinical visco-supplementation protocols, daily injections mitigate rapid drug clearance and allow drug delivery locally in the joint cavity. EGCG is nontoxic to joint tissues as shown by our histological studies and as well as other similar studies including intraarticular injections (24.30). We stained with toluidine blue to delineate articular cartilage characteristics in normal and injected joint samples. Utilizing the metachromatic properties of toluidine blue and its preferential binding to mucin, cartilage, and mast cells gave us confidence in our goal to show that our experimental approach does not alter or damage the articular cartilage It is believed that the increase in mechanical properties of AC described and demonstrated in this study is due to the crosslinking (hydrogen bonds and Van der Waals interactions) of the EGCG with the collagen proteins. This enhanced crosslinking will translate into strengthening articular cartilage and preserve the structural integrity of cartilage and finally the joint.

Polyphenols like EGCG, quercetin, and catechin have been extensively studied in preclinical research for the treatment of cancer, cardiovascular diseases, diabetes, inflammatory diseases [34]. Polyphenols have antioxidant and anti-inflammatory benefits sparking significant interest in long-term treatments [35–37]. Previous studies indicate their use in wound healing by inhibiting collagenase and the decreasing in inflammatory cytokines [38]. Previously other groups have mostly studied the role of polyphenols on the inhibition of inflammatory cytokines and MMPs in arthritis models [31, 32]. However, the present study elucidates the mechanical strengthening behavior of polyphenols relevant to articular cartilage crosslinking, a subject that has not received attention in previous studies. Further research is required to fully study both potentially beneficial and adverse effects. Excess crosslinking could induce more hardness resulting in loss of cartilage function.

It should be noted that the present study is preliminary and only biomechanical changes were measured in changes in EGCG exposed healthy cartilage. Similar experiments need to be performed in arthritic animal models and the effects of dosing; long-term effects still must be explored. For example, studies in rat osteoarthritis model are ideally suited for determining the preventive and therapeutic potential of polyphenols. Also, using these models it is possible to include a large sample size per group, providing robust data for significance analysis and moving this research in the direction of clinical fruition. Moreover, other biochemical tests are needed to measure the degree of cartilage matrix crosslinking after exposure to EGCG [39].

This study describes a method that can be used for the measurement of biomechanical properties of in-tact, hydrated articular cartilage by quasit-static nanoindentation which could be expanded further with various indentation techniques such as creep-relaxation testing or dynamic nanoindentation without the need for new sample mounting systems [39, 40]. Importantly, we believe this study provides proof-of-the-principle that it is possible to crosslink cartilage in-vivo by polyphenol such as EGCG. An observation that will have enormous clinical significance for human health and disability.

## V. Conclusion

In this study, we examined the effectiveness of a polyphenolic treatment in increasing crucial mechanical properties of healthy cartilage and connective tissue. For cartilage, we compared the findings for each leg within each subject as well as the relative increase due of treatment for all the experimental subjects compared to subjects which had no injections. Our findings, in conjunction with its established anti-inflammatory and crosslinking effects, suggest that EGCG injections could be a potential preventative measure against cartilage damage.
